# Second Language as an Exemptor from Sociocultural Norms. Emotion-Related Language Choice Revisited

**DOI:** 10.1371/journal.pone.0081225

**Published:** 2013-12-11

**Authors:** Marta Gawinkowska, Michał B. Paradowski, Michał Bilewicz

**Affiliations:** 1 Department of Psychology, University of Warsaw, Warsaw, Poland; 2 Institute of Applied Linguistics, University of Warsaw, Warsaw, Poland; Stony Brook University, United States of America

## Abstract

Bilinguals often switch languages depending on what they are saying. According to the Emotion-Related Language Choice theory, they find their second language an easier medium of conveying content which evokes strong emotions. The first language carries too much emotional power, which can be threatening for the speaker. In a covert experiment, bilingual Polish students translated texts brimming with expletives from Polish into English and vice versa. In the Polish translations, the swear word equivalents used were weaker than in the source text; in the English translations, they were stronger than in the original. These results corroborate the ERLC theory. However, the effect was only observed for ethnophaulisms, i.e. expletives directed at social groups. It turns out that the main factor triggering the language choice in bilinguals is not necessarily the different emotional power of both languages, but social and cultural norms.

## Introduction

Over centuries, language and its influence on the functioning of human beings has evoked wide interest and fascination. Whorf’s famous idea of linguistic relativity assigns to language a decisive role in affecting human thinking, resting on the assumption that speakers of different languages will perceive the world differently: “all observers are not led by the same physical evidence to the same picture of the universe, unless their linguistic backgrounds are similar” [1]: p. 214*f*. Amidst all the questions and uncertainties arising around this hypothesis, one is especially intriguing: what happens in the case of bi- and multilinguals? Do they have two (or more) ways of thinking and two (or more) sets of conceptual categories [2]? Wierzbicka [3] goes even further and claims that knowing two languages means living in two emotional worlds. Regardless of what processes are taking place in their heads, one thing is certain – bilinguals have the advantage of switching languages whenever they find it necessary, useful, or helpful.

Those who have contact with bi- or multilinguals often notice that it is easier for the latter group to express certain content in one language, while other content is more willingly conveyed in the other. What does this preference depend on? It has been noticed and proven that it is mostly emotional topics that cause slipping from one language to another. Kim and Starks [4] name this phenomenon ‘emotion-related language choice’ (ERLC) and define it as a language choice made by a bilingual person, either consciously or subconsciously, which is not conditioned by factors such as the environment (e.g. home/school/playground/workplace/pub…), but lies within their own, subjective preferences. Pavlenko [5], basing on the results of a web questionnaire for bilinguals on expressing emotions in L2, notices that words connected with emotions may be considered ‘disembodied’ in L2, whereas in L1 they seem natural, even if they are considered ‘taboo’ expressions, terms of endearment, or swear words. What is interesting, a theory postulating emotionality of L1 and artificiality of L2 can lead to two alternative conclusions: on the one hand, it should be easier for bilinguals to talk about emotion-loaded issues in the more natural L1; on the other, they may prefer L2 for that purpose, as the cultural and social norms of L1 regarding expressing emotions can be too burdensome.

### ERLC as the Way to Emotional Spontaneity

An interesting real-life proof for ERLC being triggered by the greater emotionality of L1 can be found in the diary of famous bilingual writer Eva Hoffman, who describes her dilemmas concerning the choice of language for writing her memoirs – her native Polish, or English, the language of the environment she was in at the time when the events she wanted to account for were happening. She finally chooses English, as ‘the language of the present’, but she emphasizes that it is not ‘the language of the self’, so for her writing the diary will be ‘an impersonal exercise’ [6]: p. 120*ff*. Hoffman’s feelings are consistent with the impressions of other bilingual authors, i.a. Julia Alvarez, Minfong Ho, Czesław Miłosz or Isaac Bashevis Singer, who report the inability to write in a language different from their mother tongue due to the higher emotionality of the latter.

ERLC is also present in bilinguals’ everyday lives. Scenes from multicultural marriages serve here as a good example, as it was repeatedly observed that in disagreements the spouses automatically switch to their L1 as a language more natural to express their emotions (*cf.* e.g. [7]; [8]).

The existence of ERLC has been proven in numerous studies. The overall conclusion that follows from research on language-switching behaviour is that code alternation in emotional contexts may serve a distancing function, as expressing emotions in L1 would be too burdensome and anxiety-provoking, especially for bilinguals who learned their second language later than in early childhood [9]. The theory of the distancing function of a foreign language has been recently corroborated in a study on decision-making biases [10]. What the authors call “the foreign-language effect” is a reduction of loss aversion detected while making decisions in a foreign language. The suggested explanation is greater cognitive and emotional distance evoked by the foreign language (*ibid.*). Another recent study [11] investigated the brain electrical activity in bilinguals reading pairs of words, one of them in L1 and the other in L2. As proven in previous research, bilinguals automatically activate translations in the first language when processing the meaning of words in their second language ([12]; [13]). However, the new study has shown that when the words presented in the second language are of negative emotional valence, this process is suppressed. The authors suggest that the somehow disturbing content of the words triggers inhibitory mechanisms that block access to the native language [11].

The greater emotional overtone of L1 might be connected with the first emotion experience. Altarriba [14] hypothesised that emotional expression might be more richly represented in the native language of the bilingual, because emotions were first experienced in contexts in which that vernacular was the primary language. It is presumably that context which results in L1 carrying more associations and connotations. These findings correspond with data obtained from studies on psychotherapy of bilinguals conducted in their L2 in comparison to those run in L1 ([15]–[20]). The patients investigated did not want their psychotherapy to be conducted in their native language, even when the therapist was bilingual, since using L1 during therapy would result in intense emotional reactions, whereas when L2 was used, the patients were calm and distant.

Psycholinguistic explorations, psychoanalytic case studies, and finally their own real life experiences unequivocally indicate that for bilinguals the two languages differ in their emotional impact (especially when the second language has been learnt after puberty), the first being the language of personal and emotional involvement, and the second the language of distance and detachment (or at least the language having less emotional influence on the individual). The phenomenon of ERLC is not limited to highly reflective, sensitive bilinguals who are greatly sensitive to emotional context, but to some extent is experienced by all.

### ERLC as the Way Out of the Shackles of Correctness

A particularly interesting kind of language behaviour is swearing and using taboo words. Not only does it evoke a lot of excitement and strong feelings, but it also enjoys much more attention from language learners than other vocabulary items, or grammatical structures – sometimes swear words are the only lexis they know in the language. In all languages swear and taboo words are a rather delicate and controversial field, which even native speakers may sometimes find hard to navigate. It is because the rules of using these expressions may differ depending on the context. Also attitudes towards these speech acts vary according to the situation and the speakers. In some circles and subcultures they are tolerated, whereas in other milieus and more formal contexts they may be considered unacceptable. Some countries (e.g. Australia or the United Arab Emirates) have banned swearing in public and impose fines for doing so. Taking all this into account, it is not surprising that non-native speakers face an especially challenging task entering this vocabulary domain. Even though they have acquired the rules of using strong language in their L1–their point of reference–these rules are not universal and cannot be straightforwardly transferred from one language to another. While non-native speakers acquire foreign-language swear words relatively fast [21], they usually do not have the necessary knowledge to use them in the appropriate contexts. However, regardless of their insufficient pragmatic knowledge, while swearing they still seem to prefer their Lx over L1.

This rule does not apply to highly proficient multilinguals. According to quantitative and qualitative data gathered by Dewaele [22] in an online questionnaire, multilinguals are significantly more likely to vent their anger in their L1 than in any of the languages acquired later, even though they are highly proficient in each. However, the question about the reasons for language preference presumed it to be an emotional one: *“Do swear and taboo words in your different languages have the same emotional weight for you?”* Moreover, while the quantitative data analyses showed that multilinguals definitely preferred L1 for swearing and that for them swear words in L1 carried far more emotional weight than in L2, findings based on qualitative data gathered in subsequent interviews were no longer so clear-cut. While the major reason of the respondents’ language preference was the superior emotional force of L1, some of them commented on cultural constraints which prohibited the use of swearwords. This was characteristic of participants of Arabic or Asian origin, where swearing carries strong social stigma.

In subsequent research on pentalinguals, Dewaele [23] achieved similar results. Some participants found anger expressed in their Lx “fake”. On the other hand, precisely because of the strong impact of swearing in L1, several participants (possibly due to cultural or religious beliefs) considered such linguistic behaviour taboo, which led to its avoidance; in these cases, some of them used the strategy of integrating swear words from other languages into their L1. One person reported that the language she chose for expressing anger depended on the strength of her emotions: she could express mild anger in her L2, but when it turned into real fury, she would switch to her L1. Other participants declared that they used code switching to adjust to the language of swearing used by the interlocutor.

Dewaele [23] differentiated several factors that influence code switching while swearing, the most significant of them being *language socialization* and *cultural background*. The former term refers to the frequency of using Lxs. Those who use languages other than their L1 on an everyday basis, with a wide range of interlocutors, are considered to be strongly socialized in these languages, which facilitates using them to express anger. Socialisation is closely linked to *cultural background*. Participants of Asian or Arabic origin preferred swearing in English, as it released them from the social stigma imposed on swear words in languages such as Japanese, Chinese or Arabic. This is why even when talking to their compatriots, they frequently express anger in English (*op. cit.*).

The above-described research on ERLC while swearing reveals that in the case of expletives, the language change does not have to be motivated by differences in the emotional power of both languages, but by the social norms regulating the use of such expressions.

### Ethnophaulisms

The term ‘ethnophaulism’ was introduced by Roback [24] and describes ethnic slur relating to a given out-group (social group other than that which a given person belongs to). Ethnophaulisms are part of so-called hate speech. According to Allport [25], such expressions represent a special kind of the language of prejudice; they are often rooted in ethnic conflicts. For those who use the slurs, they become a way of expressing their attitudes and feelings towards the given out-group. They may also serve as words epitomizing social stereotypes and illustrating in what ways a society looks at the group at which the slurs are directed. Even though ethnophaulisms are relatively rarely used in the presence of their targets, they frequently appear in interactions with other in-group members. Greenberg and Pyszczyński [26] proved that merely overhearing such insults automatically activates negative feelings and beliefs towards the out-group, even among egalitarian societies. In their study, the subjects were supposed to assess a debate of two attorneys – one white and the other black. In the experimental group, the experimenter made some negative remarks about the black attorney in a discriminative and racist way. Once the debate was over, the subjects–students, some of whom were declaring very modern, liberal views–were asked to judge the arguments of both attorneys. Regardless of whether the participant had expressed racist views before, under the influence of the racists remarks of the experimenter they judged the black lawyer more negatively. This proves that ethnic slurs may have a strong and implicit influence on the perception of the out-groups in question.

### Emotional Spontaneity or Social Norms as a Mechanism of ERLC? Overview of the Present Study

Based on existing literature one could derive two alternative hypotheses about the easiness of swearing in L1 and L2. According to the traditional formulation of ERLC, using more explicit emotional language in L1 should be much easier than in L2, due to the higher emotional spontaneity that bilingual language users express in their native language. Alternatively, as we discussed above, long cultural learning and socialization make expressions in L1 extremely prone to normative influences. Thus, expressing any socially unaccepted words and utterances (such as swearing) should be more difficult in L1 than in L2. In order to test whether it is norms or emotional spontaneity that have a greater impact on the willingness to swear among bilingual speakers, we designed an experimental study in which participants (Polish-English bilingual students) were asked to translate two equally offensive texts (from L1 to L2 or from L2 to L1), and to assess their offensiveness.

In order to capture the normative aspect of ERLC, we decided to confront the participants with words that are subject to higher (ethnophaulisms) and lower (general swearwords) norms of political correctness. Ethnophaulisms are a specific type of swearwords, as they relate to specific social groups and are subject to a greater number of norms and risk of disapproval. Their use means flouting political correctness and reflects not only the speaker’s lack of manners, but also their attitude towards other social groups. Therefore, in the case of such utterances, the hypothesised ERLC effect could be different than in the case of regular swear words.

Thus, the design was a 2×2×2 experiment with one between-participants factor (direction of translation: L1 to L2 vs. L2 to L1), and two within-subjects factors (rating of source material vs. rating of translated material; general swear word rating vs. ethnophaulism rating). The dependent variable was the ratings of offensiveness. If the emotional spontaneity hypothesis is true, in translations from L2 into L1 the target words should be more offensive than the source words, and the reverse should occur in L1 into L2 translations (Hypothesis 1). If the normative influence hypothesis is true, then in translations from L2 into L1 the target words should be rated as less offensive than the source words, and the reverse should occur in L1 into L2 translations (Hypothesis 2a). Additionally, if the normative influence hypothesis is true, L2 would make it easier for bilinguals to use normatively constrained words (such as ethnophaulisms), but should not influence the use of other expletives (Hypothesis 2b).

## Methods

### Participants

The participants of the present study were 61 students of Applied Linguistics and English Philology at the University of Warsaw, 11 men and 50 women at the age from 21 to 24. The subjects were picked randomly, belonged to various groups, majors and years of study. The students of both faculties were treated as Polish-English bilinguals, as their command of English is advanced and fluent. An advanced level of English had been required in the recruitment process; secondly, nearly all their courses take place in this language, and they use it on an everyday basis in various ways. Additionally, to verify their level of English the questionnaire included a measure of vocabulary knowledge.

### Procedure and Measures

The experiment is different from previous studies on swearing from the methodological point of view. To date the hypothesis that swearing is easier in L2 has been based chiefly on self-reports of bilinguals, bilingual writers and observers of bilingual people. In the present study the subjects did not know what was being investigated, so they did not take note of the fact that language change might affect their expression of emotions. There is often discrepancy between human behaviour and the perception of this behaviour, which is why the current study was designed as a covert experiment. This helped gather direct data, unbiased by the subjects’ willingness to present themselves in a positive light.

The study was conducted during two weeks of classes at the university and consisted of three stages. In the first part, the subjects were given a task: a short text which they were to translate. They were randomly divided into two groups; one translated the text from L1 Polish into L2 English, and the other from L2 English into L1 Polish (both versions of the source text are included in Appendix S1).

Under the text there was a question included to verify the subject’s knowledge of vocabulary, to make sure that it would not influence the translation. The time to do the first part was not limited, but the majority of the participants finished it in under 20 minutes.

In the next stage, the subjects were given a list of the swear words which had appeared in the *source* text. They were asked to mark the level of their offensiveness on a five-point Likert scale, ranging from 1 (“not offensive at all”) to 5 (“very offensive”). Once all the questionnaires were analysed by the experimenter, a list of all the *translated* words was created and sent to the participants via Internet, as an online survey. Their task was to rate the translated words on the same Likert-scale, ranging from 1 (“not offensive at all”) to 5 (“very offensive”). Each participant got the list with all translations, which included their translations ‘hidden’ among the translations of the others. In this way, each participant evaluated their own translation of the original words. Thus, the comparison of the level of offensiveness between the source and the target words was made by the participants themselves, which gave more insightful data than would a subjective judgment of the experimenters.

### Ethics Statement

The participants provided verbal informed consent to take part in the study. Such a form of consent is customarily used in Poland in studies on adult student samples. The consent procedures were detailed in a description submitted to the institutional review board (Ethics Committee of the Faculty of Psychology, University of Warsaw), where they were granted final approval in June 2012. The participants were granted full anonymity of the data gathered for the analyses. The final results were subsequently available to them by request (a possibility of which they had been informed before taking part in the study).

## Results

**Figure 1:**
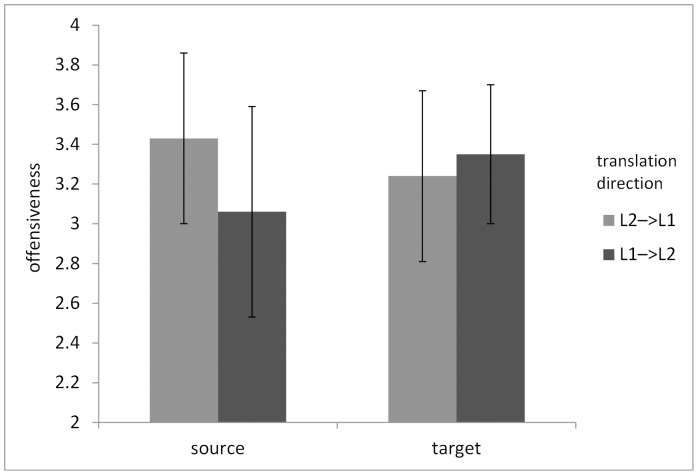
Translation direction and perceived offensiveness of expressions used.

The first step was to calculate the mean offensiveness of the source and target words in both groups. Then, using Repeated Measures ANOVA, the levels of offensiveness between the source and the target words were compared for both groups. The within-subject variable was the words (source vs. target) and the between-subject moderator was the direction of translation (L1→L2 vs. L2→L1). The dependent variable was the level of offensiveness. The analysis showed a significant interaction between the source and target words and the direction of translation, *F*(1,59) = 7.88; *p*<.01; eta^2^ = .12. The post-hoc analyses showed that in the translations into English the source words were significantly less offensive (M = 3.06, SD = .53) than their translations (M = 3.35, SD = .35), *p*<.05. The results are presented in Figure 1.

To verify Hypothesis 2, the words were split into “ethnophaulisms” and “other swear words”. Ethnophaulisms included all and only those swear words from the text that referred to social or ethnic groups. The interaction was investigated with the use of Repeated Measures ANOVA. The within-subject variables were i) the words (source vs. target) and ii) the type of insults (ethnophaulisms vs. other swear words); the between-subject moderator was the direction of translation (L1→L2 vs. L2→L1). The dependent variable was the level of offensiveness. The analysis showed a significant interaction between the source and target words, direction of translation, and type of words, *F*(1,59) = 59, *p*<.01; eta^2^ = .16. It turned out that the significant differences in offensiveness between the source and the target words appear only in the case of ethnophaulisms. The post-hoc analyses showed that:

- in the translations from L1 into L2, the target ethnophaulisms were more offensive (M = 2.79, SD = .42) than their source words (M = 2.43, SD = .59), *p*<.05;
- in the translations from L2 into L1, the target ethnophaulisms were less offensive (M = 2.12, SD = .53) than their source words (M = 2.87, SD = .51), *p*<.01.

**Figure 2:**
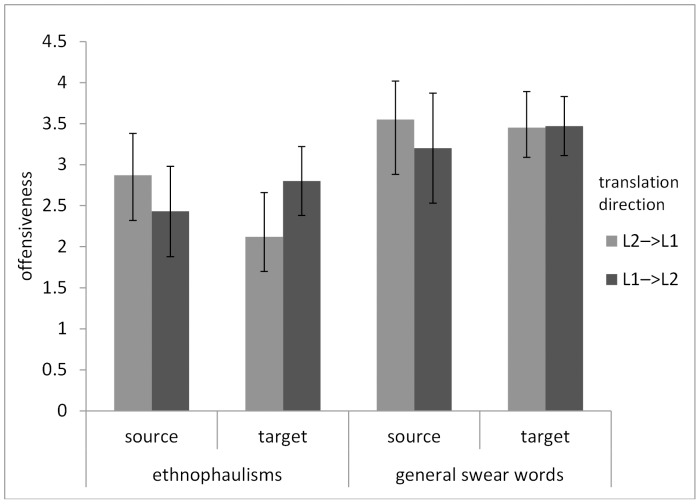
Ethnophaulisms vs. ‘general’ swear words – comparison of offensiveness differences.

These effects did not appear in the case of other swear words. The results are presented in Figure 2.

Due to their explicit language which may have the potential of offending some readers, the most extreme examples of the effects observed are presented separately in Translation Samples S1 and S2.

Translation Samples S1 includes the Polish translations (L2→L1), in which the original swear words got softened. The relevant expressions are in bold. In these translations some of the swear words were omitted, intentionally or not, while a lot of them were softened. In the last translation there are virtually no swear words, as none of the insulting words used there can be considered an expletive. It is the most extreme case, which, however, may result from the fact that this person generally does not swear or considers it unacceptable.

Interestingly, there was a similar case among the English translations (L1→L2), in which some of the swear words were softened, at odds with Hypothesis 1 (reproduced in point 1 of Translation Samples S2). It can be a premise that some people feel too limited by social norms and they are not able to breach them.

In the remaining translations into English, the swear words supplied by the subjects were more offensive than in the source texts. In some cases not only were the existing swear words strengthened, but also some more expletives were added. Some representative examples of the English translations are presented in point 2 of Translation Samples S2, with the relevant expressions in bold.

## Discussion

The ERLC theory in the case of swearing implicates two different hypotheses: it is easier to swear in a foreign language either because it distances the speaker emotionally from the expressed content or it exempts them from the social constraints concerning using swear words. The research confirmed the normative influence hypothesis. Bilinguals find it easier to swear and to offend out-groups in L2. Even though the participants were bilingual, the results of the study can reasonably be extrapolated to all people who know a foreign language at a communicative level. It transpires that the foreign language exempts us from our own or socially imposed norms and limitations and makes us more prone to swearing and offending others.

On the one hand, our findings are consistent with those cited in the literature overview. On the other, they bring a fresh perspective into research on language choice in bilinguals. They prove the distancing function of L2 claimed by Bond & Lai [9] and observed in the psychotherapy of bilingual patients (i.a. [15]; [16]), and are consistent with the results of Dewaele’s studies on swearing in multilinguals ([22], [23]). However, it must be underlined that whereas previous investigations in this area concentrated on establishing which language bilinguals prefer to express emotional content, we have revealed what exactly influences their choice. In the research cited above, no interpretation other than the emotional hypothesis had been taken into consideration, except for Dewaele’s studies ([22], [23]), in which some of the participants reported that using swear words in their L1 was taboo and that L2 allowed them to escape the social constraints. Additionally, Dewaele highlighted factors which might affect the use of swearwords in multilinguals and pointed out that the *cultural background* is closely linked to swearing in L2, as it released the speakers from the social stigma of using expletives. Still, this finding was not contrasted with the emotional factor. Such contrasting was performed in our study and, as the effect was observed only in the case of normatively protected expletives, it turns out that it is social norms and limitations that really motivates bilinguals to swear in L2. It thus appears that if the emotion-laden words are at the same level of social acceptance, there should be no difference for bilinguals as for in which language to express them.

In relation to the ERLC study, the question arises whether the results were not caused by the context of the experiment–it was conducted in Poland by a Polish experimenter. Could it have been the fact that the subjects were in their homeland–the place where L1 is spoken – that made them too constrained by the applicable norms and as a result they softened the words when translating into Polish? Given that the experiment was carried out at university in a class which would normally take place in the English language, this concern can be dismissed. Still, it is worth investigating whether, had the study been conducted in a different context, the subjects would have felt equally or more comfortable swearing in their L1.

There is also the problem of different constraints present in the Polish and English cultures, which are conveyed through the language. One may argue that in English-speaking countries it is more common to hear swear words in public, e.g. in the media. On the other hand, political correctness emanating from the Anglo-Saxon culture is definitely more rooted in English than in Polish. The problem was explored in a study by Bilewicz and Bocheńska [27], who measured the influence of language on prejudice towards black people. They assumed that bilinguals switching into a more ‘politically correct language’ would express their stereotypical beliefs to a lesser extent than in a language which is ‘more tolerant’. The subject were Polish-French bilingual students, with L2 French. Polish discourse was thought to convey values and judgments in a more explicit way than French, which was supposed to be more implicit and subtle. Indeed the results showed that those participants who were asked to complete the prejudice questionnaire in L2 French ascribed more humanity to black people. However, the effect was observed almost only for the ‘cognitive’ part of prejudice (cognitive schema or stereotype), and there was actually a marginally higher ‘affective’ prejudice (the attitudinal factor, which is the affective reaction to the object of prejudice; [25]) in this group – they declared minimally less positive emotions toward the group than those who responded in L1 Polish (*op. cit.*). Thus, it seems that even if L1 and L2 may differ in their level of tolerance for swear words and ethnophaulisms, this kind of emotional content may always be more easily expressed in L2. The implications for future research should include conducting more experiments of this kind, but trying to make subjects produce texts spontaneously, preferably to express their own views, so that it will be more certain that the production of the material required activating emotional processes. It is also worth considering carrying out the experiments in the contexts of two languages and comparing the results.

The normative influence discovery undoubtedly opens new perspective in the research on language choice in bilinguals. It proves that there is something more to it than the straightforward emotional distancing explanation. It implies that the use of language does not depend on the speaker’s inner experiences and feelings, but, at least in the case of swearing, rather on the social reality in which they function.

## Supporting Information

Appendix S1 **Stimulus materials (source texts in L1 Polish and L2 English).**
(RTF)

Translation samples S1 **L2 → L1.**
(RTF)

Translation samples S2 **L1 → L2.**
(RTF)
