# Supplementary material for: Second Language as an Exemptor from Sociocultural Norms. Emotion-Related Language Choice Revisited
Source: PLoS One. 2013 Dec 11;8(12):e81225. doi: 10.1371/journal.pone.0081225 (PMC3859501; doi:10.1371/journal.pone.0081225)
Supplement: Appendix S1 — Stimulus materials (source texts in L1 Polish and L2 English). (RTF) [file pone.0081225.s001.rtf]

1.	Poni¿ej znajduje siê wypowiedŸ zaczerpniêta z forum internetowego za³o¿onego przez Polaków mieszkaj¹cych w Anglii
„Chcia³bym coœ sprostowaæ - Eminem jest bia³y i uda³o mu siê, wiêc nic dziwnego, ¿e czarni go nienawidz¹. Pieprzeni murzyni wygl¹daj¹ jak gówno. Eminem podobno Polski nie lubi, a to gówno prawda, nigdy nie wypowiedzia³ siê negatywnie o Polakach, to cholerni geje z Polski puœcili tak¹ durn¹ plotê, a to dlatego, ¿e wielokrotnie wyœmiewa³ siê w piosenkach z homoseksualistów i ¯ydów. Mogliby siê kurwa zamkn¹æ, wkurwiaj¹ mnie ci debile. Nie mówiê, ¿e go za to lubiê - jak widaæ korzysta z wolnoœci s³owa, teraz siê o tym du¿o mówi, ¿e unia, prawa cz³owieka, wolnoœæ s³owa, zajebiœcie. Tylko ¿e w unii rz¹dz¹ pojebani faszyœci i ¿ydki. Jeœli przyg³upi czarnuch skacz¹c jak ma³pa obra¿a w swoich debilnych piosenkach innych ludzi, to faktycznie, korzysta z wolnoœci s³owa... Wiecie co wam powiem? Eminem to pieprzony æpun, skurwiel, który wymyœli³ sobie, ¿e jak nie ma talentu, to zarobi kasê na rzucaniu gównem, tak w³aœnie myœlê.”

2.	Below you can see a post taken from a forum created by Poles living in the UK
“I'd like to make one thing clear - Eminem is white and he made it, so no wonder black people hate him. Fuckin niggers look like crap. People say he doesn't like Poland and its bullshit, he's never said anything bad about Polish people, damn gays from Poland spread this crap rumour because Eminem is always making fun of homosexuals and Jews in his songs. They should shut the fuck up, they're pissing me off, morons. I don't say I like him because of that - apparently he's just using the freedom of speech, you hear a lot about it these days, the Union, human rights, freedom of speech fucking awesome. But the Union is ruled by screwed up fascists and kikes. If a dumb nigger can jump around like a monkey and insult other people in his imbecile songs then yes, he's just using his freedom of speech... You know what? Eminem is a fuckin drug addict, bastard, who isn't really gifted so he's trying to make money by throwing shit, that's what I say.”
