# Supplementary material for: Second Language as an Exemptor from Sociocultural Norms. Emotion-Related Language Choice Revisited
Source: PLoS One. 2013 Dec 11;8(12):e81225. doi: 10.1371/journal.pone.0081225 (PMC3859501; doi:10.1371/journal.pone.0081225)
Supplement: Translation samples S1 — L2 → L1. (RTF) [file pone.0081225.s002.rtf]

HM12: Chcia³bym wyjaœniæ jedn¹ rzecz – Eminem jest bia³y i jemu siê uda³o, wiêc trudno siê dziwiæ, ¿e czarni go nienawidz¹. Pieprzeni czarni wygl¹daj¹ jak gówno. Mówi¹, ¿e on nie lubi Polski, ale to ……… , nigdy nie powiedzia³ nic z³ego o Polakach, pieprzeni Polacy sami rozpowszechniaj¹ tê gównian¹ plotkê, poniewa¿ Eminem w swoich piosenkach zawsze naœmiewa siê z homoseksualistów i ¯ydów. Powinni siê zamkn¹æ, wkurzaj¹ mnie, idioci. Nie twierdzê, ¿e lubiê go z tego powodu – on najwyraŸniej korzysta tylko wolnoœci s³owa, tyle siê o tym dzisiaj s³yszy, Unia, prawa cz³owieka, wolnoœæ s³owa, zajebiœcie cudownie. Ale Uni¹ kieruj¹ popaprani faszyœci. Je¿eli g³upi murzyn mo¿e skakaæ jak ma³pa i obra¿aæ innych w swoich idiotycznych piosenkach to jasne, on korzysta tylko z wolnoœci s³owa. Wiecie co? Eminem jest pieprzonym narkomanem, idiot¹, który wcale nie jest uzdolniony i stara siê zarobiæ wciskaj¹c gówno, tak twierdzê.

JK09: Chcia³bym wyjaœniæ jedn¹ rzecz – Eminem jest bia³y i jemu siê uda³o, wiêc nic dziwnego, ¿e czarni go nienawidz¹. Pieprzone murzyny wygl¹daj¹ jak upoœledzeni. Ludzie mówi¹, ¿e on nie lubi Polski, ale to gówno prawda, nigdy nie powiedzia³ nic z³ego na temat Polaków. G³upi kolesie z Polski rozsiewaj¹ tê pog³oskê, bo Eminem zawsze naœmiewa siê z homoseksualistów i ¯ydów w swoich piosenkach……………………………………………… Nie twierdzê, ¿e go z tego powodu lubiê – przecie¿ u¿ywa jedynie wolnoœci s³owa, o której tak czêsto siê mówi, Unia, prawa cz³owieka, prawo g³osu – wspaniale kurwa. Ale Unia rz¹dzona jest przez popieprzonych faszystów i ………… Jeœli g³upi murzyn mo¿e skakaæ jak ma³pa i obra¿aæ innych ludzi w swoich niedorzecznych piosenkach to tak, u¿ywa w³aœnie wolnoœci g³osu. I wiecie co? Eminem jest pieprzonym narkomanem, który nie jest w ogóle utalentowany, wiêc próbuje byæ s³awny przez rzucanie miêsem, to moje zdanie.

£O28: Chcia³bym wyjaœniæ jedno – Eminem jest bia³y, a mu siê uda³o, nic dziwnego, ¿e czarni go nienawidz¹. Mówi siê, ¿e on nie lubi Polski, a to bzdura. Eminem nigdy nie powiedzia³ nic z³ego o Polakach, pieprzeni geje z Polski rozsiewaj¹ te gówniane plotki, bo on w swoich piosenkach zawsze naœmiewa siê z homoseksualistów i ¯ydów. Niech siê lepiej zamkn¹ te debile, wkurzaj¹ mnie. Nie mówiê, ¿e to dlatego go lubiê. On po prostu korzysta z wolnoœci s³owa, du¿o teraz siê o tym s³yszy, o Unii, prawach cz³owieka, wolnoœci s³owa, po prostu kurwa wspaniale. Ale Uni¹ rz¹dz¹ pieprzeni faszyœci i peda³y. Jeœli têpy czarnuch mo¿e skakaæ jak ma³pa i obra¿aæ ludzi w swoich piosenkach to tak, korzysta z wolnoœci s³owa. Wiecie co? Eminem to narkoman, drañ bez talentu wiêc próbuje robiæ kasê rzucaj¹c miêsem, wed³ug mnie.

IK19: Chcia³bym zauwa¿yæ jedno – Eminem jest bia³y i osi¹gn¹³ to, wiêc nie dziwcie siê dlaczego czarni go nienawidz¹. Niemili czarnoskórzy ubieraj¹ siê niechlujnie. Mówi siê, ¿e Eminem nie lubi Polski co nie jest prawd¹, on nigdy nie wypowiada³ siê negatywnie na temat Polaków, to homoseksualiœci z Polski roznosz¹ pog³oski, gdy¿ on w swoich tekstach zawsze naœmiewa siê z ¯ydów i gejów/homoseksualistów. Powinni siê zamkn¹æ, bardzo mnie denerwuj¹, g³¹by/idioci. Nie lubiê go jednak za to – w zasadzie pos³uguje siê tylko wolnoœci¹ wypowiedzi, czêsto siê o niej dzisiaj s³yszy, Unia, prawa cz³owieka, wolnoœæ wypowiedzi, s¹ niesamowite. Unia jednak jest prowadzona przez bandê nienormalnych faszystów i homoseksualistów. Jeœli ma³o wyedukowany czarnoskóry skacze jak ma³pa, obra¿a ludzi swoimi g³upimi tekstami, u¿ywa tylko wolnoœci wypowiedzi. Wiecie co? Eminem jest niewyedukowanym narkomanem, ³ajdakiem, nie bêd¹c wystarczaj¹co utalentowanym obrzuca ludzi b³otem, tym w³aœnie jest.
