# Supplementary material for: Second Language as an Exemptor from Sociocultural Norms. Emotion-Related Language Choice Revisited
Source: PLoS One. 2013 Dec 11;8(12):e81225. doi: 10.1371/journal.pone.0081225 (PMC3859501; doi:10.1371/journal.pone.0081225)
Supplement: Translation samples S2 — L1 → L2. (RTF) [file pone.0081225.s003.rtf]

1.
PK02: I would like to put something clear – Eminem is white and he managed, so nothing strange that blacks hate him. Fuck*** blacks look like ***. Eminem doesn't like Poland, but it is not true, he never said anything bad about Poles, gays from Poland said it because he sang negatively about gays and Jews. They could shut up, they annoy me, idiots. I don't mean that I like him – he says what he likes, he can. Nowadays everybody says about freedom of speech etc… If a stupid black behaves like an ape and offends others in his songs, then indeed, uses freedom of speech. Do you know what I want to say? Eminem is a fuck*** rubbish, who imagined that without talent he can do the cash by offending people.

2.
UB25: I'd like to make something clear – Eminem is white, no wonder that black people hate him. Those fucking niggers look like shit. People say Eminem doesn't like Poland, but it's not fucking true – he never said a bad words about Poles. These were the damn faggots from Poland who spread that shitty rumour and that's only because he picked on them and the Jews in many of his songs. They could just shut the fuck up, these morons really piss me off. I'm not saying I don't like him – he's just speaking his mind – now it's a very popular topic: EU, human rights, freedom of speech – that's fucking awesome. The only thing is that the EU is governed by fucked-up fascist and Jews. If a stupid nigger who is jumping like a monkey says shit about other people in his songs, is this what you call the freedom of speech? You know what? Eminem is a fucking junkie, a motherfucker who figured out that he's gonna make cheese on talking shit about others, as he's got no talent – that's what I think.
DC27: I'd like to make things clear – Eminem is white and he made it, so it's no wonder that the blacks hate him. Fucking niggers look like the shit. They say Eminem doesn't like Poland, but it's bullshit, he never said anything wrong bout Poles, it were those fucking faggots from Poland that spread the gossip and it's because he often made fun of homos and Jews in his songs. They might just shut their fucking mouth, they piss me off like shit, these assholes. I'm not saying it makes me like that guy – apparently he enjoys the freedom of speech, you hear people talking about that a lot these days, the Union thing, the human rights thing fucking great. It's just that it's fucked up fascists and Jews who govern the Union. When a retarded nigger jumps like a monkey and offends other ppl in his dumb songs then he's actually enjoying the freedom of speech. You know what? Eminem is a fuckin' junkie, a motherfucker who thinks that if you ain't got a talent you can make money on throwing shit at people, that's what I think.
PA11: I'd like to set sth straight – Eminem is white and he managed, so no wonder niggers hate him. Fucking negroes look like shit. Eminem supposedly doesn't like Poland, but it's bullshit, he never said a bad word 'bout Poles, it's just the fucking faggots from Poland who claimed such shit, and that's 'cause he made fun of fags and Jews in his songs. They should just shut the fuck up, those fucking morons piss me off. I'm not saying I like him for that – he just uses freedom of speech, there's a lot on about this, european union, human rights, freedom of speech, fuckin' awesome. But EU is ruled by fuckin' fascists and Jews. If a dumb monkey-nigger offends people in his songs then yeah, he sure does. You know what I say? Eminem is a fucking junkie, motherfucker who thought that since he has no talent he'll just make cash for throwing shit around, that's what I think.
WS02: I would like to say it simply – Eminem is white and he made it. So that is the reason why blacks hate him. Fucking blacks who look like shit. Probably Eminem doesn't like Poland. That's a bullshit. He has never said anything rude about Poles. This fucking gossip was spread by fucking gays from Poland, because Eminem laughed at homosexuals and Jews. They could finally shut up, they make me angry. I'm not saying I like him for that – he just uses the freedom of speech. Nowadays it is said a lot about European Union, human rights and freedom of speech, fucking great. But European Union is ruled by fucking fascists and Jews. If fucking stupid negro jumps like monkey and offends other people in his stupid songs, it's all right because he is using his freedom of speech. Do you know what I say you? Eminem is a fucking fixer, fucking ass who imagined himself that he, without a talent, can make cash by throwing his shit at people. That's what I think.
